# A Novel Implantable Glaucoma Valve Using Ferrofluid

**DOI:** 10.1371/journal.pone.0067404

**Published:** 2013-06-28

**Authors:** Eleftherios I. Paschalis, James Chodosh, Ralph A. Sperling, Borja Salvador-Culla, Claes Dohlman

**Affiliations:** 1 Massachusetts Eye and Ear Infirmary, Harvard Medical School, Boston, Massachusetts, United States of America; 2 School of Engineering and Applied Sciences, Harvard University, Cambridge, Massachusetts, United States of America; Faculty of Medicine University of Leipzig, Germany

## Abstract

**Purpose:**

To present a novel design of an implantable glaucoma valve based on ferrofluidic nanoparticles and to compare it with a well-established FDA approved valve.

**Setting:**

Massachusetts Eye & Ear Infirmary, Boston, USA.

**Methods:**

A glaucoma valve was designed using soft lithography techniques utilizing a water-immiscible magnetic fluid (ferrofluid) as a pressure-sensitive barrier to aqueous flow. Two rare earth micro magnets were used to calibrate the opening and closing pressure. In-vitro flow measurements were performed to characterize the valve and to compare it to Ahmed™ glaucoma valve. The reliability and predictability of the new valve was verified by pressure/flow measurements over a period of three months and X-ray diffraction (XRD) analysis over a period of eight weeks. *In vivo* assessment was performed in three rabbits.

**Results:**

In the *in vitro* experiments, the opening and closing pressures of the valve were 10 and 7 mmHg, respectively. The measured flow/pressure response was linearly proportional and reproducible over a period of three months (1.8 µl/min at 12 mmHg; 4.3 µl/min at 16 mmHg; 7.6 µl/min at 21 mmHg). X-ray diffraction analysis did not show oxidization of the ferrofluid when exposed to water or air. Preliminary *in vivo* results suggest that the valve is biocompatible and can control the intraocular pressure in rabbits.

**Conclusions:**

The proposed valve utilizes ferrofluid as passive, tunable constriction element to provide highly predictable opening and closing pressures while maintaining ocular tone. The ferrofluid maintained its magnetic properties in the aqueous environment and provided linear flow to pressure response. Our in-vitro tests showed reliable and reproducible results over a study period of three months. Preliminary *in-vivo* results were very promising and currently more thorough investigation of this device is underway.

## Introduction

Glaucoma is a group of diseases that cause loss of vision due to progressive degeneration of the retinal ganglion cells. [1], [2] The pathogenesis of the disease is still unknown, but it is well established that lowering the IOP can slow down the progression of the disease. [3], [4] To date, IOP regulation is the primary target in glaucoma management and is obtained either pharmaceutically (topical or systemic pressure-lowering drugs) or surgically (trabecular filtration surgery or drainage device implantation).

Among the various surgical procedures, drainage devices have gained popularity mostly due to their ease of use, their efficacy in IOP reduction and due to the growing concerns about late complications associated with standard filtering surgery. [5], [6].

Most glaucoma drainage devices are fitted with a soft silicone tube end that is inserted in the anterior chamber (AC) through a scleral fistula that shunts aqueous humor (AH) to an end plate with flow resistance. In plated valves, the end plate is surgically placed under the conjunctiva in the equatorial region of the globe. Variations on plate size and valve/shunt architectures have been described previously.[6]–[9] These variations were shown to affect both the IOP as well as the overall function of these devices. The main drawback in the current designs is that they rely on the additional flow resistance provided by the conjunctival plate encapsulation. [5], [9] As a consequence, the majority of post-operative complications are associated with the encapsulation process resulting in early hypotony or later ocular hypertension. [6], [10] Other factors that may contribute to this process include ocular injuries, inflammation and autoimmune syndromes. [9] Other non-valved shunts, such as the Express™ are also placed under a partial-thickness scleral flap which carries similar complication rate of bleb encapsulation as the current valves. [11].

As a result, there is a clear need for a valve that can reliably and predictably regulate the IOP without relying on the additional resistance from encapsulation (bleb). It will also be advantageous to have a device designed for external placement of the outlet tip (plate) while, most important, providing a good closing pressure thus preventing hypotony. Such valve arrangement could potentially address the complication of plate encapsulation, especially in keratoprosthesis patients with cicatrizing ocular diseases. [9] The possibility of placing the outlet tip at the lower lid fornix could provide accessibility for observation and replacement under a slit-lamp during a routine clinic encounter.

Ferrofluids are made of magnetic nanoparticles suspended in an inert, non-magnetic carrier fluid. Due to their small size, typically 10 to 100 nm in diameter, the particles are subject to Brownian motion. Small magnetite (Fe_3_O_4_) particles consist of a single magnetic domain and exhibit super-paramagnetic properties, i.e. a drag force along field gradients. To prevent aggregation, the particles are coated with a surfactant that is compatible with the carrier fluid they are suspended in. [12] Hence, in the presence of a static magnetic field, the interfacial force will cause the ferrofluid to conform to its boundaries. Thus, a ferrofluid in a capillary tube can act as an on/off valve to flow through capillary blockade. In order to generate an aqueous flow barrier, both the carrier fluid and the surfactant must be non-polar to provide water immiscibility. Then, a permanent magnet can be used to hold the ferrofluid in the capillary and a secondary magnet can be used to adjust the force required to form the capillary barrier. When the pressure exerted on the ferrofluid by the liquid exceeds the magnetic force between the secondary magnet and the ferrofluid, the barrier is broken and flow can initiate. Typically, the distance between the secondary magnet and the ferrofluid determines the strength of this barrier as will be described in more detail below.

Within this work, we present a novel, easily replaceable, ferromagnetic valved tube capable of providing pressure regulation without the necessity of subconjunctival encapsulation. We describe the design architecture and characterize its performance in an in-vitro manometric setup. In addition we compare the performance of the new ferrovalve with an FDA approved glaucoma valve (Ahmed™). Finally, we undertake characterization of the ferrofluid under long-term water and air exposure using X-Ray diffraction (XRD) analysis.

## Methods

### Design of the Ferrovalve

The design of the valve was based on soft lithography techniques using polydimethylsiloxane (PDMS), a very well characterized and biocompatible polymer. Sylgard 184 (Dow Corning Corporation, Midland, MI, USA) flexible silicon elastomer was used with a base to curing agent ratio of 10∶1 by weight, to prepare the liquid pre-polymer.

**Figure 1:**
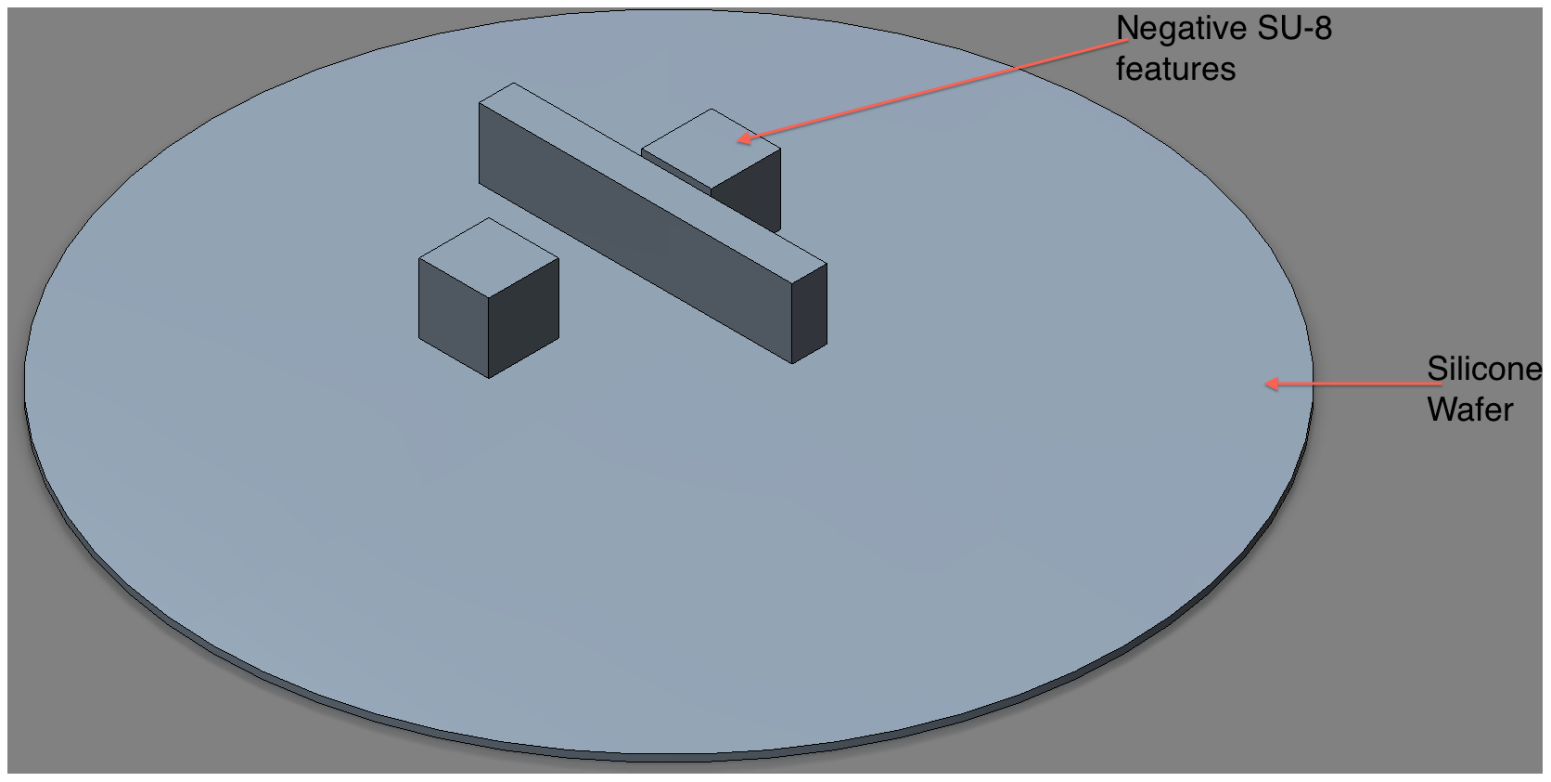
A silicon wafer with 100 µm height features of negative SU-8 photoresist. The design was used as a master mold for the valve.D.

The design of the valve involved 4 steps:

In step 1, a master mold was created using permanent epoxy negative photoresist SU-8 3050 (MicroChem corporation, Newton, MA, USA). The SU-8 was spin-coated on a 3 inches silicon wafer and exposed to UV light using a photo mask with the desire features (1000 rpm/100 µm feature size). After developing the master, PDMS was poured over the master and cured by baking at 65°C overnight (Figure 1).

In step 2, a capillary tube of clear fused quartz (VitroCom Mountain Lakes, NJ, USA) with inner diameter (ID) 300 micrometers and outer diameter (OD) 400 micrometers was silanized by flushing it with a PEG-silane solution ([methoxy(polyethyleneoxy)propyl]trichlorosilane, Gelest SIM6492.66). The capillary was then cut to 5.5 mm length using a diamond blade and inserted into a soft silicone tube of 300 micrometers ID (VWR International 60985–700 0.30 mm ID and 0.61 mm OD). The tube was then mounted on the PDMS mold (from step 1) with two 1/16″ cubic rare earth magnets (Nd_2_Fe_14_B, K&J Magnetics, Inc., Jamison, PA, USA). The PDMS was placed on a silicon wafer in a petri dish and PDMS pre-polymer was poured and cured at 65° C over night to embed the capillary tube and magnets in PDMS.

In step 3, the device was cut out from the bulk PDMS (step 2) and trimmed to its desired shape and size (2.8×4.7×2.7 mm in Length x Width x Height). One millimeter length of silicon tube was left on both sides for tube connection. A stainless steel tube of 400 micrometer OD and 12 mm length (New England Small Tube, Litchfield, NH, USA) was used to interconnect the valve with a longer soft silicone tube (VWR International 60985–700). The connection was secured with PDMS.

**Figure 2:**
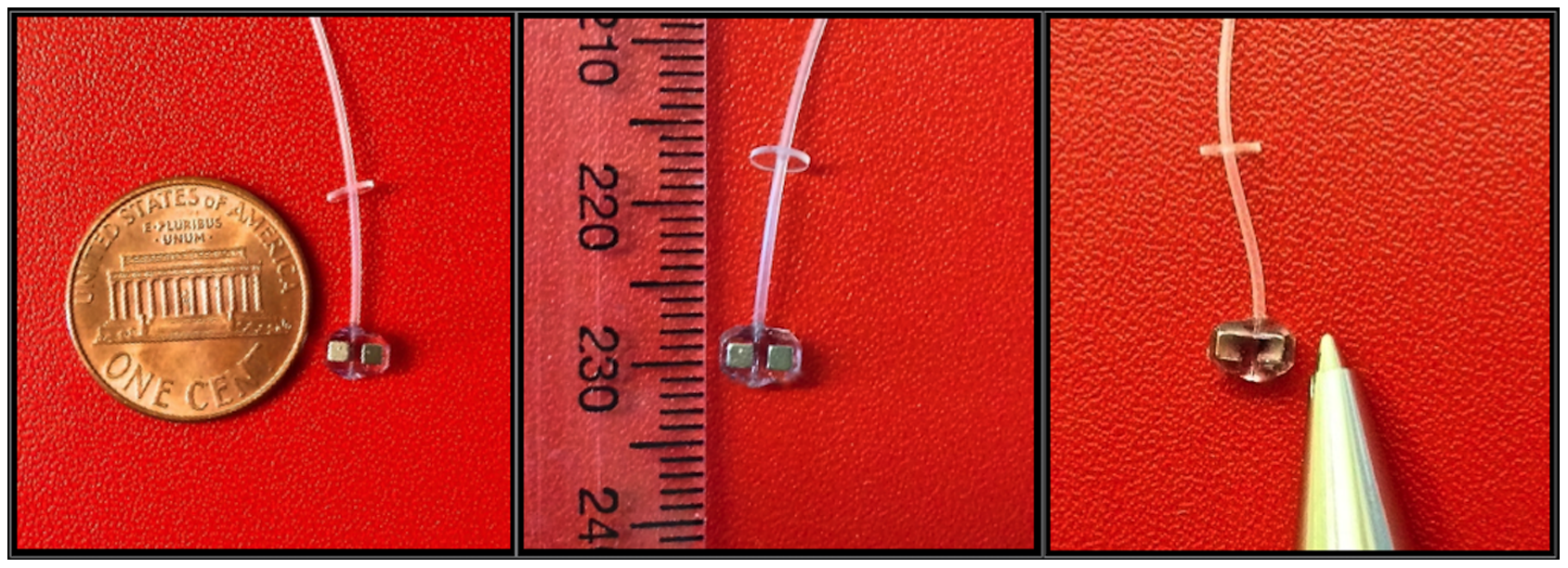
A prototype implantable ferromagnetic glaucoma valve (ferrovalve). Size comparison with one US cent coin (left), the tip of a pen (center) and a ruler of 1 cm scale (right). The size of the valve is 2.8×4.7×2.7 mm in Length x Width x Height. You can also distinguish the circular PDMS peg on the tube for scleral suturing.

In step 4, a small amount of ferrofluid (∼0.1 µL), generously provided by Ferrotec corporation (Bedford, NH, USA), was introduced into the capillary using a 30 gauge needle. The ferrofluid was made of 10 nm monodispersed Fe_3_O_4_ particles suspended in a fluorocarbon carrier oil. The ferrofluid has a viscosity of 367 cP and maximum magnetization of 404 Gauss (NF 3914). The ferrofluid was securely fixed by the magnetic force of the two micro magnets, such that the primary micro magnet next to the tube holds the ferrofluid in place while the secondary acts as a pressure regulator (Figure 2).

### Calibration and Characterization of the Ferrovalve

**Figure 3:**
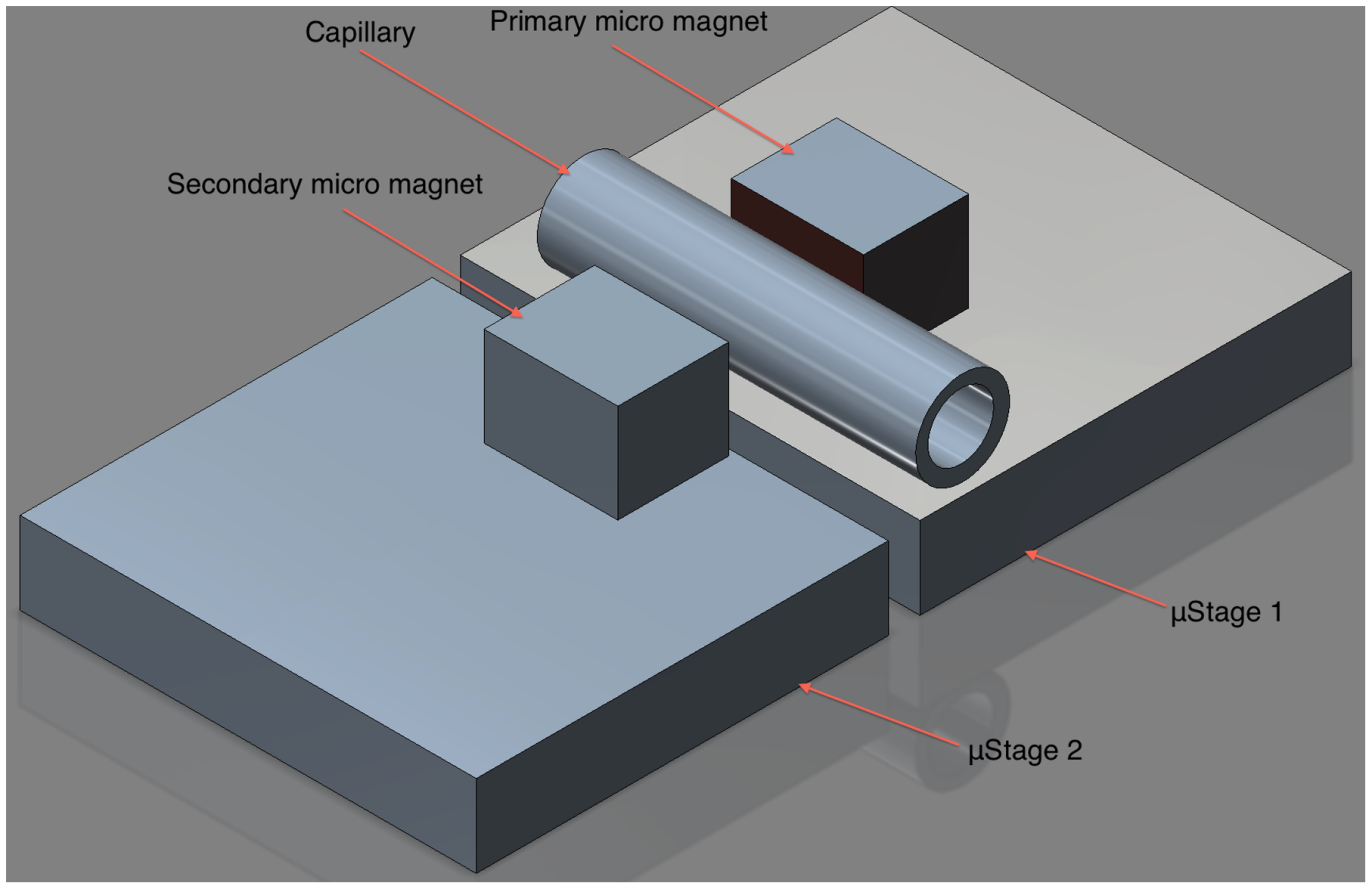
Valve calibration using two movable micro stages. The first stage contains the tube with a micro magnet providing strong magnetic field on the ferrofluid. The second contains a micro magnet which position can be adjusted to provide weaker magnetic field on the ferrofluid.

Before building the final prototype of the ferrovalve with all components embedded in PDMS, we set up a modular system in which the magnets could be moved in respect to the capillary tube containing the ferrofluid, in order to study the pressure/flow characteristics of the design. The setup was as follows:

The capillary valve was placed on a micro stage (Thorlabs, Newton, NJ, USA) and connected to a reservoir containing distilled water (dH_2_O) via a 40 cm silicone tube (VWR International 60985–700 0.30 mm ID and 0.61 mm OD). The primary micro magnet was placed adjacent to the capillary valve with the north magnetic pole towards the direction of the ferrofluid. A second micro magnet was placed on a separate micro stage with its south magnetic pole facing the primary magnet (Figure 3).

The primary magnet served as a fixation of the droplet by providing the highest magnetic attraction on the ferrofluid, while the secondary magnet placed at the opposite distal end served to control the opening and closing pressures of the valve. In this way, the configuration of the PDMS device is replicated in a discrete fashion that allows adjusting the distance between the second magnet and the ferrofluid inside the capillary tube.

Each magnet had a surface field of 5754 Gauss providing a pulling force of 68 g. The maximum energy product ((BH)max) of each magnet was about 42 MGauss·Oersted (according to the specifications of the manufacturer).

For experiments aiming at physiologically relevant pressures, the distance of the secondary magnet was adjusted to provide flow opening at 10 mmHg of pressure.

**Figure 4:**
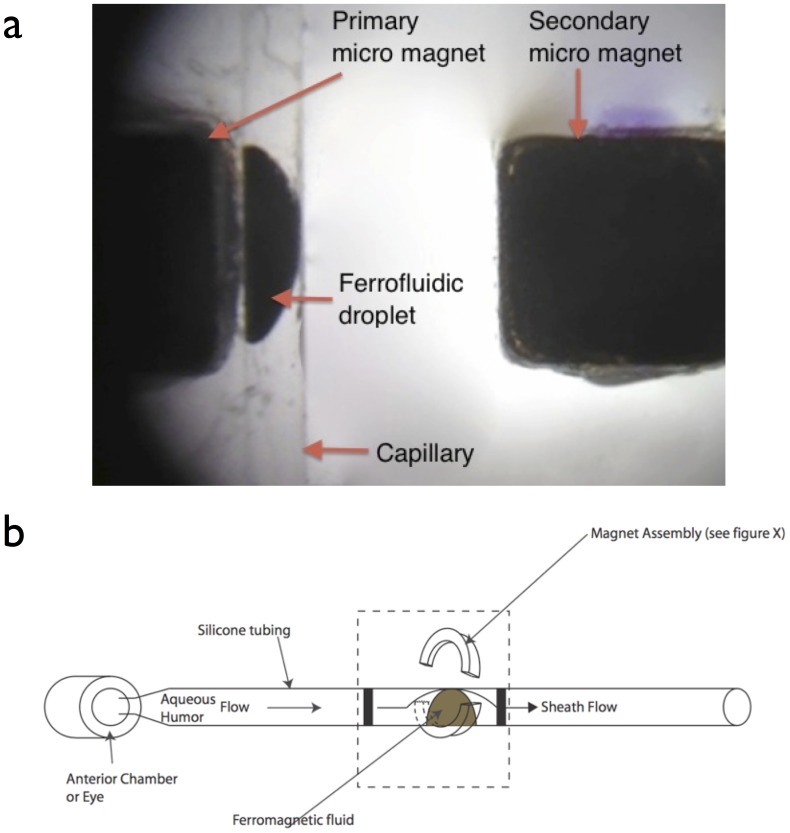
Representation of the ferrofluidic valve architecture. (a) Enlarged photograph of the valve. An adjustable micro magnet (left), the capillary with the ferrofluid (center), and the fixed micro magnet (right). (b)Schematic diagram of the ferromagnetic valve.

A small amount of ferrofluid (0.1 µL) was introduced in the capillary using a custom-made glass needle. Both magnets provided bidirectional magnetic attraction to the ferrofluid with the secondary having less magnetic effect than the primary due to its greater distance from the capillary (figure 4). Hence, the magnetic field lines going from one magnet to the other keep the ferrofluid in place against the flow through the capillary. Only a high-enough pressure provides the necessary force to displace the ferrofluid sufficiently to form a channel and allow liquid flow through the capillary.

The dH_2_O reservoir was elevated to a defined height to generate a hydrostatic pressure difference, as calculated using the following formula:

P = ρgh.

[P: pressure, ρ: density of liquid, g = 9.8 N/kg and the value equals gravitational acceleration, h: height of liquid in meters.].

The hydrostatic pressure was converted to millimeters of mercury pressure by substituting the liquid density of water (1.00 g/mL) by the density of Hg (13.55 g/mL). Pressure and flow measurements were carried out continuously for 3 months.

Pressure calibration was achieved by adjusting the distance of the secondary micro-magnet to obtain an opening and closing pressure of 10 and 7 mmHg, respectively. The secondary micro-magnet was offset horizontally to compensate for the ferrofluid bending towards the flow direction. Various test pressures were set by adjusting the height of the dH_2_O reservoir.

### Characterization of the Ferrofluid

To study potential oxidation of the Fe_3_O_4_ nanoparticles to Fe_2_O_3_, the ferrofluid was characterized using X-Ray diffraction (XRD) crystallography. Samples of the ferrofluid were exposed to either water or air for 8 weeks and chemical analysis was obtained in order to evaluate the oxidization on the nanoparticles. The nanoparticles in the ferrofluid were investigated by large angle X-ray diffraction (XRD, Scintag XDS2000). The peaks of 2θ obtained by the X-ray diffractogram were correlated using the International Center for Diffraction Data and crystal structure and composition was identified. In addition, the ferrofluid was assessed under an optical microscope over the course of the experiment in order to evaluate its integrity.

**Figure 5:**
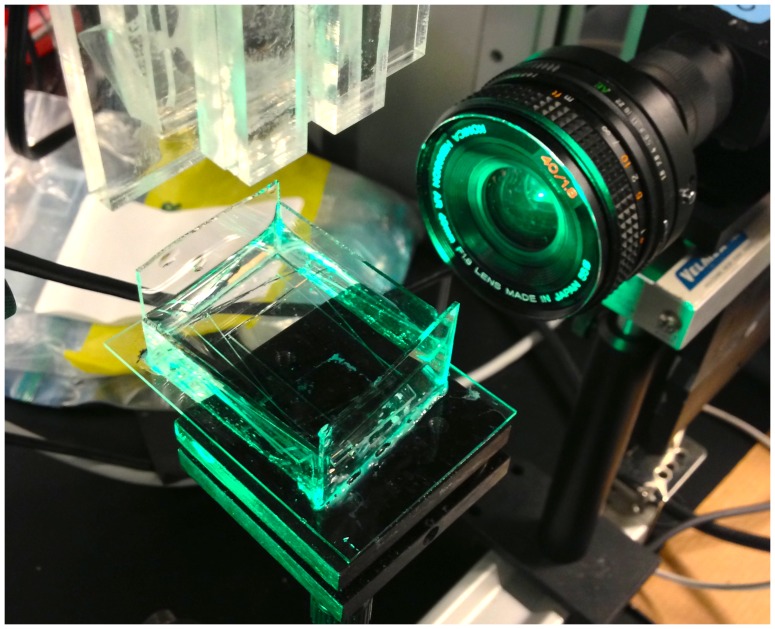
A computer-controlled CCD camera with green LED background illumination and a water cuvette for measuring the contact angle of the ferrofluid droplet on coated and non-coated glass slide.

The interaction between the ferrofluid and the glass capillary was assessed with contact angle and adhesion measurements. A computer-controlled CCD camera (Sony XCD-V50) with green LED background illumination was used to measure the contact angle of a ferrofluid droplet on hydrophilic and hydrophobic coated glass slide in order to assess the surface interaction between the glass and the ferrofluid. The glass slides we silanized with either a perfluorosilane (hydrophobic) or PEG-silane (hydrophilic), while one slide was left non-coated. All slides were thoroughly cleaned by rinsing them several times with ethanol and water, and blown dry with compressed filtered air. Contact angle measurements were obtained in air and water interface using a custom-made glass cuvette (figure 5). The adhesion of the ferrofluid to the various substrates was probed using a jet of pressurized air in an attempt to blow away the ferrofluid droplet from the substrate. The results were optically captured as remaining deposits of ferrofluid on substrates. This qualitative assessment provided insights to the surface interaction between the different coatings and the ferrofluid.

Further evaluation of the glass coatings was undertaken with contact angle measurements of water and Fluorinert FC77 oil droplets in air and immersed under water.

### Ahmed™ valve

In order to compare the pressure and flow measurements of the ferrovalve with the current standard of care, we used the commercially available, well established, and FDA-approved Ahmed™ Glaucoma Valve (New World Medical Inc., Rancho Cucamonga, CA). The Ahmed™ PF-7 silicone valve was connected to the same dH_2_O reservoir, as previously described. Flow vs. pressure measurements were recorded and the opening and closing pressure was determined in similar fashion as for the ferrovalve.

### *In vivo* Experiments

The use of animals for this study was approved by the Animal Care Committee of the Massachusetts Eye and Ear Infirmary, and all animal procedures were performed in accordance with the ARVO Statement for the use of Animals in Ophthalmic and Vision Research and the National Institute of Health Guidance for the Care and Use of Laboratory Animals.

**Figure 6:**
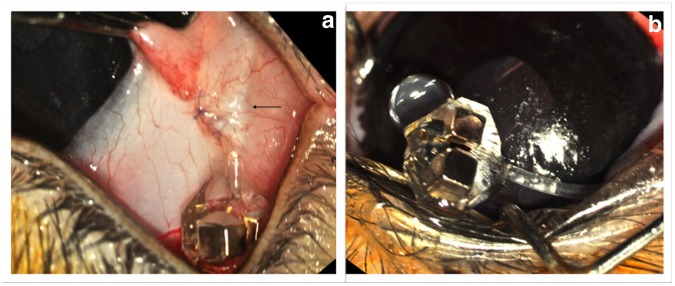
Implantation of the ferromagnetic valve in a rabbit eye. Black arrow indicates the sub-conjunctival tunnel over the valve tubing. A) The housing remains exterior at the bottom of the lower lid fornix. B) Flow of aqueous humor is shown *in vivo*.

Implantation was performed in three NZ White/NZ Red crossed rabbits (male, weighing 4.3–4.5 Kg). The ferrovalve housing was connected with a 35 mm of silicone tubing (inner diameter = 300 µm; outer diameter = 610 µm) that was trimmed during the surgery and placed at the lower lid fornix temporally. The tubing was then inserted beneath the conjunctiva and was led up to the supra-temporal limbus. The tip was then inserted into the posterior chamber of the eye using a 25 G needle. A small peg, previously attached to the tubing, was anchored to the sclera with two 10-0 Nylon sutures, for stability. Thus, the tubing extended subconjunctivally to enter the eye from a point in the lower fornix. The ferrovalve housing remained exterior to the conjunctiva at the inferior lid fornix (Figure 6).

The animals were treated daily with topical Polytrim (polymyxin B/trimethoprim, Allergan, Inc., USA) and Pred-forte (prednisolone acetate 1%, Allergan, Inc., USA), and followed for two weeks. Eyes were assessed for inflammation and infection and daily IOP measurements were performed.

## Results

### Pressure Calibration

**Figure 7:**
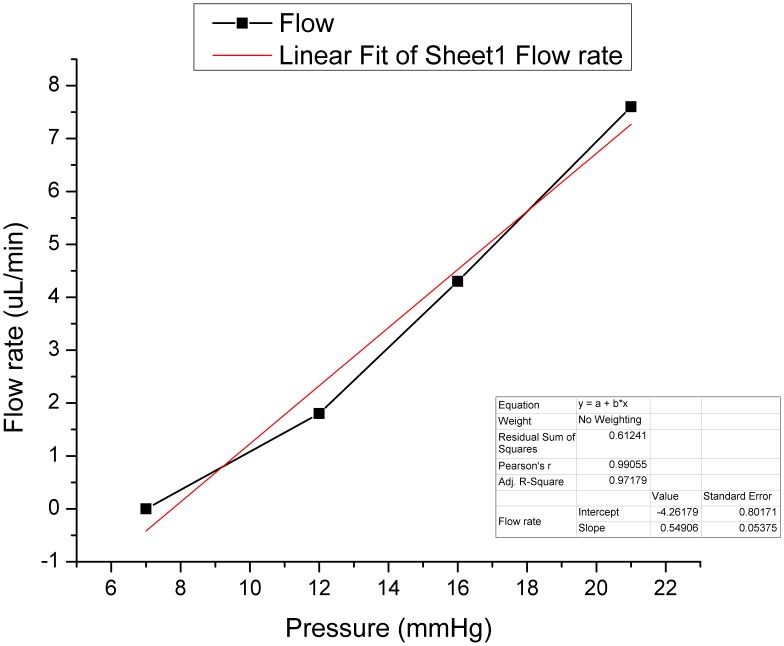
Linearity of the pressure/flow relationship of the ferromagnetic valve (Adj. R2 = 0.971). The valve provides a flow rate of 1 µL/min at 10 mmHg of pressure.

A water-immiscible ferrofluid droplet of 0.1 µL volume was placed in the tube. The primary magnet was placed in contact with the tube subsection containing the ferromagnetic droplet and both were mounted on a microstage (µStage 1). A second magnet was placed on a different microstage (µStage 2), moving vertically and opposite to the first (µStage 1). By adjusting the horizontal spacing between the µStage 1 and µStage 2 to 500 µm, the magnetic field of the secondary magnet on the ferrofluid droplet was weakened, thus obtaining an opening and closing pressure of 10 and 7 mmHg, respectively. This pressure calibration was maintained over the entire duration of the experiment (3 months) with small variations that resulted to pressure drift of less than 0.5 mmHg. Experimental results at pressure of 7 mmHg over a period of one week showed no flow or significant leak. Similarly, over a period of 30 days the pressure was varied between 7, 12, 16, and 21 mmHg. The resulting opening and closing pressure was maintained as initially calibrated (open: 10 mmHg/close: 7 mmHg) with a small variability of ±0.5 mmHg. The flow/pressure response of the valve showed increase of flow at higher pressures (1.8 µl/min at 12 mmHg; 4.3 µl/min at 16 mmHg; 7.6 µl/min at 21 mmHg). Flow variations were clinically insignificant during the study period (±0.2 µL/min at 12 mmHg). Analyzing the data collected during the 3 months of experimentation, a linear response was found between flow and pressure (Adjusted R^2^ = 0.971) (figure 7). The results represent the mean flow rate at 7, 12, 16 and 21 mmHg of pressure over a period of three months under varying pressure conditions.

At 12.5 mmHg of pressure the outflow facility achieved was equal to the aqueous humor (AH) production rate in normal eyes (∼2.5 µL/min). These results suggest that as long as the valve functions under the pre-defined specifications, the pressure in the eye cannot exceed the upper threshold of 12.5 mmHg, even if the valve filters the total AH volume produced per minute.

However, presuming some remaining functionality of the trabecular meshwork and the uveo-scleral pathway, which are the principal AH draining sites in the normal eye, this pressure could easily be reduced to 10 mmHg. Even at this pressure, the valve would be able to drain approximately 50% of the AH produced per minute. [13].

**Figure 8:**
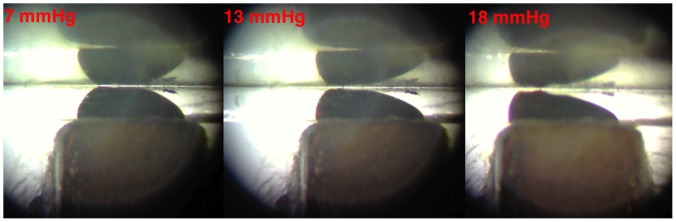
The valve mechanism at different pressures. Closed at 7 mmHg (left), open at 13 mmHg (center), open at 18 mmHg providing increased flow rate (right).

The mechanism of opening and closing is presented in figure 8, which illustrates the shape change in the water immiscible meniscus of ferrofluid under the influence of various pressures.

### XRD Results

The ability of the magnetic Fe_3_O_4_ nanoparticles to withstand oxidization was assessed over an 8 weeks period of exposure to water and air. This experiment serves as a qualitative predictor of the performance, integrity and durability of the ferrovalve.

**Figure 9:**
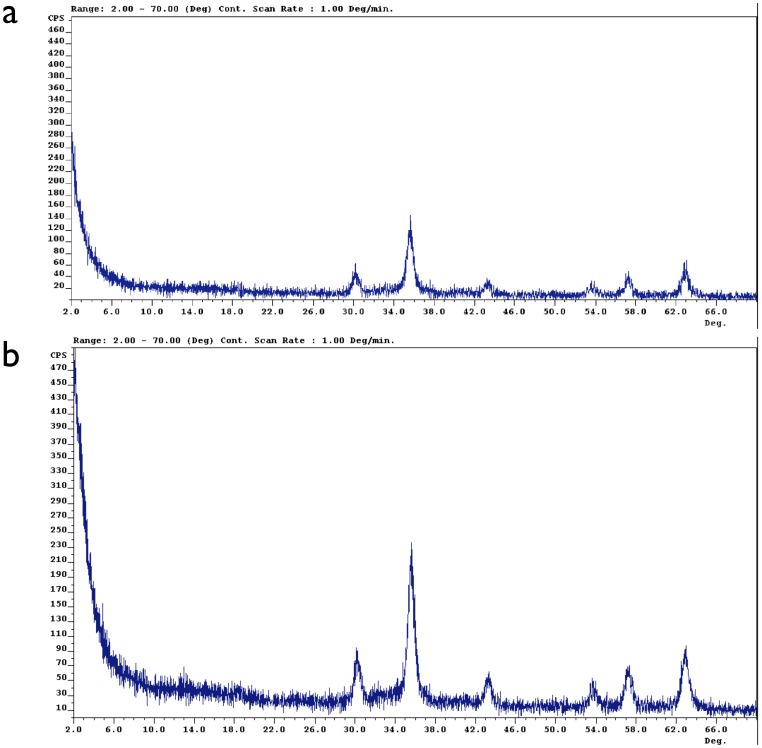
Stability analysis of magnetite Fe3O4 nano-particles exposed to water and air exposure using X-ray diffraction analysis. (a) Top figure: X-ray diffraction analysis (XRD) of the ferrofluid pre-exposure to water and air. The 2θ peaks at 30° and 35.5° are characteristic of magnetite Fe_3_O_4_. (b) Bottom figure: X-ray diffraction analysis (XRD) of the ferrofluid post-exposure to water and air for 8 weeks. The 2θ peaks at 30° and 35.5° are characteristic of magnetite Fe_3_O_4_. No oxidization was observed on the ferromagnetic nano-particles.

X-ray diffraction (XRD) experiments were performed to characterize the stability of the Fe_3_O_4_ particles suspended in a fluorocarbon-based carrier fluid. The ferrofluid was exposed to water and oxygen at room temperature. XRD results before exposure, figure 9(a), and at 8 weeks post-exposure, figure 9(b), showed that the 2θ peaks (the peaks at approximately 30° and 35.5°) are characteristic of magnetite (Fe_3_O_4_), suggesting that the ferrofluid remained chemically stable. Similar results were previously obtained by the authors in additional experiments testing the oxidization of the ferrofluid following three months of flow/pressure exposure (data not shown).

**Figure 10:**
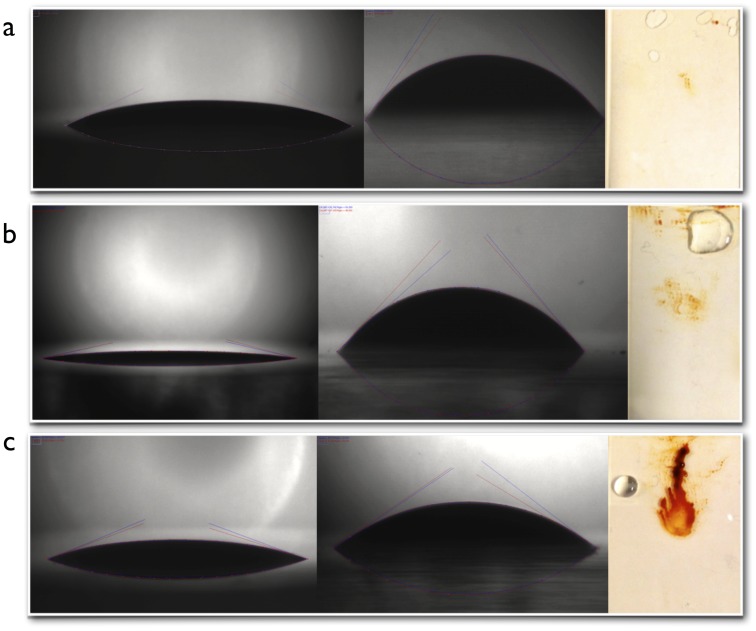
Contact angle measurements of the ferrofluid on different substrates in air (left) and water (center). Images on the right show the different substrates after the drop of ferrofluid has been blown off with compressed air. (a) Plain glass (non-coated) contact angle and adhesion measurements. (b) PEG-silane (hydrophilic) contact angle and adhesion measurements. (c) Fluorosilane (hydrophobic coating) contact angle and adhesion measurements.

The contact angle of the ferrofluid in air was φ_air_≈ 25° for plain glass; 13° for PEG-silane coated glass and 19° for fluorosilane coated glass. However, the angle measurements in water interface showed variable results between the coatings with the highest angle achieved by the plain glass (φ_water_≈ 52°), and PEG (φ_water_≈ 48°) and the lowest with the fluorosilane coating (φ_water_≈ 32°), figure 10 (a,b).

**Figure 11:**
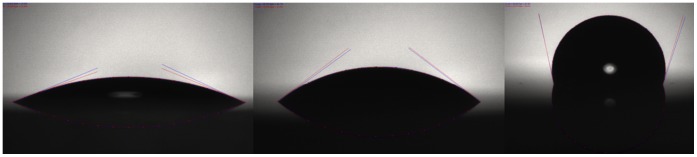
Contact angle measurements of a water droplet on non-coated (left), PEG-silane coated (center) and fluorosilane coated glass slides (right).

The contact angle of water was φ_air_≈ 20° for the non-coated glass, φ_air_≈ 37° for the PEG-silane coated glass and φ_air_≈ 104° for the fluorosilane coated glass suggesting that the non-coated glass is more hydrophilic than the PEG-silane coated glass, figure 11.

In a different experiment using Fluorinert (FC-77) oil, simulating the carrier fluid of the ferrofluid, the contact angle in air was φ_air_≈ 5° for the non-coated glass, φ_air_≈ 9° for the PEG-coated and φ_air_≈ 26° for the fluorosilane coated glass, while in water was φ_water_≈ 143° for the non-coated glass, φ_water_≈ 124° for the PEG-silane coated and φ_water_≈ 38° for the fluorosilane coated glass.

Increase in hydrophilicity led to increase in contact angle measurements. However, plain glass exhibited higher contact angle measurements in water compared to the PEG coated glass.

The adhesion assessment of the ferrofluid with the glass was achieved using a jet of pressurized air to blow away the ferrofluid droplet from the glass surface. This has led to the qualitative characterization of the interaction between the ferrofluid with the glass surfaces. The fluorosilane-coated glass exhibited the highest adhesive interaction, followed by the PEG and with the plain glass showing the least interaction, figure 9 (a–c).

### Ahmed™ Valve

In a series of repeated pressure/flow measurements with the Ahmed valve, no closing pressure was recordable in an in-vitro set-up with the outlet tip exposed to atmospheric pressure. Flow was interrupted only when the dH_2_O container was completely empty. Observation under the microscope revealed a continuous sheath flow between the two parallel membranes that form the valve mechanism. We also observed that when the accumulated water at the outlet tip of the Ahmed valve was drained using a filter paper, the water film between the membranes dewetted, which caused the membranes to come into contact and interrupt the flow. The closing pressure at that point was measured to be 2.5 mmHg with a variation of +/−2 mmHg.

### *In vivo* Experiments

Implantation of the valve was successfully performed without any complications in all three cases. The mean time of surgery was approximately 40 minutes. The circular PDMS peg resulted in a good fixation of the tube to the sclera. During the 2 weeks of follow-up, there were no signs of infection or inflammation other than a brief period of irritation right after surgery. The measurement of IOP was performed daily in both eyes, with the mean values in the valve-implanted eye (11.8±2 mmHg), significantly lower than the contralateral control eye (14±3 mmHg), (P<0.0001; paired sample t-test). These values were in agreement (+/−2 mmHg) with those obtained in vitro.

At the last follow-up, two weeks after surgery, a continuous flow was still noticed at the outlet tip of the valve, demonstrating functionality during the time of post implantation observation.

## Discussion

As previously described, pressure below 5 mmHg (“hypotony”) may lead to many ocular complications and may result in loss of vision as well as loss of the eye. [14] It is essential that any implantable valve should maintain a critical closing pressure to avoid hypotony. Available AH drainage devices, such as the Ahmed™ Glaucoma Valve and the Baerveldt® glaucoma implant rely to various degrees on the extra resistance provided by the subconjunctival encapsulation of the valve plate in order to prevent hypotony. However, this encapsulation process is often delayed and unpredictable due to variable degree of fibrosis of the bleb. As a result, the post operative management is often complicated by early hypotony that can cause influx of blood into the anterior chamber, retinal bleeding and detachment and later ocular hypertension that can accelerate the glaucoma progression. Even though the Ahmed™ valve is not usually associated with early post-operative hypotony, our in-vitro results suggest that it requires subconjunctival implantation to achieve a closing pressure. No closing pressure was measured when the outlet tip was exposed to atmospheric pressure. The water between the valve membranes provide a continuous sheath flow. We attributed the lack of closing pressure to water surface tension preventing the membranes from complete de-wetting. During our experiments we noticed that when the accumulated water at the outlet tip of the valve was manually removed, a closing pressure was achieved. We believe that the dewetting of the water film between the two membranes caused the membranes to adhere. However, under normal operating conditions of continuous flow there was no closing pressure, suggesting that the Ahmed™ valve should not be used extraocularly.

Efforts have been made to shunt AH to atmospheric pressure via a modified Ahmed™ valve with a distal tube to the maxillary sinus or to the lower lid fornix. [9] The incidence of endophthalmitis was extremely low and equal to standard trabeculectomy (0.7% on the basis of 145 cumulative shunt years). In fact, the only endophthalmitis encountered among 34 patients implanted with such external valve shunt occurred after a tooth root abscess and without signs of infection around the shunt. [9] However, maintaining a closing pressure was impossible, rendering the eye susceptible to hypotony and its associated visual consequences. In contrast, the ferromagnetic valve, as described in this study, could be placed externally in the lower lid fornix via a tube and provide the desired closing pressure as well as an opening pressure. The unidirectional flow of AH reduces the possibility of endophthalmitis even when the valve is extra ocular.

In this manuscript we present and characterize a novel glaucoma valve based on ferromagnetic nanoparticles. In the design of this valve we adhered to the general principles of simplicity and efficacy. Our work demonstrates in-vitro reproducible closing and opening pressures of the valve with a hysteresis of only 3 mmHg between opening and closing pressure.

The excellent pressure response was attributed to the ferrofluid’s ability of the ferrofluid to radically alter its geometrical characteristics at the event of pressure stimuli.

This work was undertaken in an attempt to address the limitations of the commercially available devices, which rely on encapsulation of the valve’s outlet to limit flow. Our promising preliminary data suggest that further in-vivo evaluation should be undertaken.

### Ferromagnetic Calibration

A custom-made ferrofluid, consisting of water-immiscible ferromagnetic nanoparticles that are dispersed in a fluorinated oil as carrier liquid, was used for the design of the valve. Two permanent NdFeB rare-earth micro magnets were used to provide non-conduct adjustment of the ferrofluid droplet. The primary magnet was placed next to the tube sub-section containing the ferrofluid to hold the droplet from moving with the flow, while the secondary magnet was placed opposite to the primary, adjusting the pressure required to bend the droplet and initiate flow. The bending force was evaluated using liquid flow at various pressures. The ferromagnetic valve provided flow occlusion at a pressure of 7 mmHg and flow initiation at a pressure of 10 mmHg.

These results suggest that external implantation of the outlet tip of the valve, such as at the lower lid fornix is possible without the need of encapsulation for additional flow resistance. External placement of the valve has the advantage of device accessibility for direct observation and replacement when needed.

### Ferrofluid Biocompatibility

Medical devices and their component materials may leak compounds or have surface characteristics that can produce undesirable effects when used clinically. However, based on the FDA, ISO and JMHLW guidelines, the general testing framework in the assessment of a device biocompatibility is different between implantable and external communicating devices. Nevertheless, any implantable device must comply with the safety and biocompatibility regulations. The scope of this work was the in-vitro evaluation of the device, thus literature research was undertaken to assess the biocompatibility and safety of the materials used.

Magnetic nanoparticles, such as Fe_3_O_4_, have been extensively used in magnetic field induced localized hyperthermia for the treatment of cancer,[15]–[17] for high contrast magnetic resonance Imaging (MRI) and for iron deficiency due to hemodialysis. [18], [19] No adverse cytotoxic effects have been reported in intra-venous (IV) administration. Similar nanoparticles were used in this study.

Perfluoroalkylpolyether (PFPE) is used as carrier fluid and surfactant to disperse the Fe_3_O_4_ magnetic nanoparticles. In laboratory animal tests, as reported in the manufacturer’s catalogue, the carrier liquid was found to have a low order of actuate oral activity. The LD50 dose in rats was greater than 25,000 mg per kg of body weight (data provided by the manufacturer).

There is no known hazard from short term skin exposure or inhalation at room temperature, as true for other fluorocarbon oils that are used in biomedical applications, such as during retinal detachment surgery. [20], [21].

Our new valve is designed such that the outlet tip will be placed externally in the lower lid fornix. This will minimize the potential ocular contents exposure to ferrofluid by the extra protection afforded by the conjunctiva and tear film. In addition, the total ferrofluid volume in the valve is approximately 0.1 µL, which is a minute volume compared to the volume used in retinal detachment surgeries (average 5 mL). We, therefore, expect good biocompatibility of the device and steps towards in-vivo assessment of the valve have already been taken by the authors.

### Flow Measurements

The flow capacity of this valve was studied in this paper under different pressure regimes. The flow capacity of the valve was considered as the second most important parameter in the in-vitro evaluation. IOP is a function of the outflow capacity versus the AH production rate at a given pressure. At a given IOP the valve should be able to facilitate the volume of AH that is produced in the eye per unit time.

### Contact Angle Measurements

The variability in the water contact angle measurements was attributed to the wettability of the glass slide. When the slides were submerged into water, water molecules compete with the fluorocarbon molecules in the wettability of the glass. As glass is hydrophilic, water can wet the surface well and thus reduce the contact area of the ferrofluid which results in a more compact droplet. However, the resulting contact angle on the PEG-silane coated slide was smaller than the plain glass, which led to the assessment of the ferrofluid interaction with the coating. The adhesion measurements of the ferrofluid on the glass slides showed less interaction between the ferrofluid and the plain glass slide. The results were confirmed using water and FC77 droplets on non-coated and PEG-silane coated glass. Despite of the increased hydrophilicity of the PEG-silane, non-coated glass hydrophilicity was better, providing less adhesion of the ferrofluid on the glass. These results suggest the non-coated glass is likely to perform better due to the lower interaction with the ferrofluid.

Reducing the interaction of the ferrofluid with the capillary walls was shown to provide better ferrofluid adhesion under high flow rates, smoother bending of the ferrofluid under pressure and less break-up in the event of micro air bubbles flowing through the capillary which can cause gradual loss of the ferrofluid material.

### XRD Results

Oxidization of ferromagnetic particles is a topotactical reaction where magnetite (Fe_3_O_4_) can become maghemite (γ-Fe_2_O_3_) upon further oxidation. Such transformation could decrease the saturation magnetization of the ferromagnetic particles, changing the magnetic characteristics of the ferrofluid and permanently affecting its reliability of the valve. [17].

### *In vivo* Experiments

Implantation of the ferrovalve in 3 rabbit eyes showed predictable IOP regulation for at least 2 weeks. No inflammation, keratitis, or other adverse events were noted, indicating that the valve was well tolerated and biocompatible. In a similar arrangement in 34 patients operated between 2001–2005, [9] no discomfort was noted by placing a modified Ahmed™ valve at the lower lid fornix. However, the study was stopped due to hypotony. The limited discomfort of such an arrangement is attributed to the lack of lower lid movement during blinking. The implantation of the ferrovalve was technically uncomplicated. The use of non-magnetic instruments is recommended to this surgery. The circular PDMS peg used around the tube provided good fixation to the sclera with additional sealing of the scleral tunnel. These preliminary *in vivo* results were encouraging, and a larger *in vivo* study with longer follow up is underway.

### Study Limitations

One of the limitations of this device is the incompatibility with static magnetic field produced either by permanent magnet of by flow of direct current (DC), such as in magnetic resonant imaging (MRI) systems. However, this is well known issue with other implantable devices, such as pacemakers, implantable neurostimulators, cochlear implants and osteosynthesis which face similar limitations. In the scenario were MRI is inevitable, the valve can be temporarily removed using a tight suture or a plug around the tube to prevent AH leakage. More options will be evaluated in future studies. Walk-through metal detectors, such as those used in airports do not generate static magnetic fields since they operate in alternate current (AC). However, Eddy currents can be generated but are insignificant for the valve since it does not contain active electronic elements. A broader clinical study incorporating more animals is necessary to address the aforementioned limitations. Nevertheless, preliminary *in vivo* results encourage further exploration in this direction.

### Conclusion

In this paper we describe the design and characteristics of a novel glaucoma valve. The proposed device utilizes a ferrofluid to provide highly predictable opening and closing pressures while maintaining ocular tone. Moreover, our valve is suitable for external implantation, which would facilitate maintenance and replacement in a non-invasive fashion. Preliminary *in vivo* results using rabbits suggest good tolerability and regulation of the IOP.
